# Cervical Necrotising Fasciitis Leading to Critical Airway Compromise: A Case Report of Successful Airway Management With Awake Fibreoptic Intubation

**DOI:** 10.7759/cureus.57126

**Published:** 2024-03-28

**Authors:** Hannan Chaudery, Paul Efthymiou, Stefan R Cozma

**Affiliations:** 1 Anaesthesia, King's College Hospital NHS Foundation Trust, London, GBR

**Keywords:** predicted difficult airway, difficult airway management, awake fiberoptic intubation, airway compromise, cervical necrotising fasciitis

## Abstract

A 56-year-old female patient with a history of breast cancer, anxiety, and depression developed rapid-onset cervical necrotising fasciitis following a fall at home where she sustained multiple rib fractures and lacerations. The case highlights the challenge of managing a rapidly progressing airway obstruction and the successful management of the patient's condition with awake fibreoptic intubation and subsequent surgical intervention.

## Introduction

Cervical necrotising fasciitis (CNF) is a severe, life-threatening soft tissue infection. The condition is characterised by rapidly spreading inflammation and necrosis of the fascia, subcutaneous tissues, and occasionally, the skin [1]. While the condition is most commonly caused by polymicrobial infection, it can also arise from infection by a single pathogen, as highlighted in this case. 

CNF can result in airway obstruction due to rapid swelling and inflammation in the neck, thus necessitating immediate medical intervention [2]. The disease is often associated with significant morbidity and mortality, primarily due to the rapid progression of symptoms, resulting in complete airway compromise, and the potential for systemic toxicity. 

This case report highlights the development of CNF in a patient who sustained lacerations from a fall at home, where one small posterior scalp laceration became colonised. It underscores the critical importance of thorough primary, secondary, and tertiary surveys in trauma patients and diligent management and dressing of even small wounds to prevent severe complications such as hospital-acquired infections. 

The case further underscores the need for prompt recognition of the signs and symptoms of CNF and immediate intervention to manage airway obstruction, illustrating the successful use of awake fibreoptic intubation (AFOI) in this context.

This report was also presented at the Difficult Airway Society (DAS) Conference as a poster presentation in November 2023.

## Case presentation

A 56-year-old female patient with a history of breast cancer (treated with surgery, radiotherapy and tamoxifen), hypertension, anxiety, and depression presented to the hospital following a fall at home. She had sustained several rib fractures (Rib Fracture Score 5) and small lacerations to the back and head. On day two of admission, erector spinae block and catheter insertion were performed for analgesia, and an infusion of 0.1% bupivacaine was commenced which was effective. 

**Figure 1:**
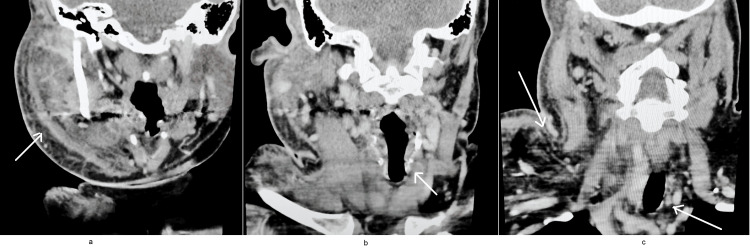
CT Neck a: Diffuse soft tissue swelling and inflammation of the right side of the face b: Swelling and inflammation of the right side of the neck with resulting tracheal deviation to the left c: Soft tissue swelling and inflammatory changes extending down to the upper chest, with distal tracheal deviation to the left.

On day 8 of admission at 2 am, she was noted to have swelling on the right side of her face and neck in the posterior triangle region starting from the retro auricular area. By 4 am, this had progressed further, the area was exquisitely tender and erythematous, and she had developed a pyrexia of 38.5 degrees. A CT scan of the neck with intravenous contrast was conducted (Figure 1) which was reported as "non-specific diffuse soft tissue inflammation in the right side of the neck which suggests the possibility of evolving cellulitis in keeping with likely Ludwig's angina". The scan further demonstrated mild-moderate tracheal deviation to the left as a result of the inflammation and swelling. 

The patient was in extremis, with swelling now including the upper chest area. Mouth opening was less than 1.5 centimetres; she was unable to swallow her own saliva and was experiencing difficulty in breathing. The appearance and progression of symptoms led to a provisional diagnosis of Ludwig's angina. 

Management

The patient was urgently referred to the anaesthesia team, and immediate management was commenced: 15 litres of oxygen via a non-rebreather mask (to maintain a SpO_2_ of 94%), 100mg of intravenous hydrocortisone, and 1mg of nebulised adrenaline to temporize the airway. The patient was emergently transferred to the theatre, where an AFOI was performed. The airway was topicalized with 1%, 2% and 4% lidocaine and xylocaine. 40mg of propofol was administered when the flexible bronchoscope passed through the glottis into the trachea. The airway was secured on the first pass, and the patient was anaesthetized with a further 120mg of Propofol and paralyzed with 50mg of Rocuronium. Full AAGBI monitoring was available at all times.

She was transferred to the Intensive Care Unit (ICU) and was reviewed by the ENT and Oral and Maxillofacial Surgery teams. A small area of necrosis was noted on the posterior scalp around one of the lacerations sustained during the initial fall. The patient returned to the theatre the next day for wide debridement and excision of necrotic and infected tissues in the scalp and neck. Tissue cultures were positive for gram-negative rods, confirmed to be E. coli. Based on the clinical presentation, imaging, and tissue cultures, the final diagnosis was CNF.

Follow-up and outcomes 

The patient developed E. coli bacteraemia requiring vasopressor support, had a prolonged ICU stay, and was later tracheostomised, after which she was successfully weaned from the ventilator, decannulated after three months, and discharged with no long-term sequelae. 

## Discussion

This case highlights the importance of prompt diagnosis and management of severe soft tissue infections like CNF that can cause rapid airway obstruction. The development of the condition following a fall that caused multiple lacerations emphasises the need for thorough primary, secondary, and tertiary surveys in trauma patients and appropriate management, cleaning and dressing of even small wounds to prevent hospital-acquired infections. 

The successful management of this patient's airway with AFOI emphasises its value in situations where the airway is threatened by rapid tissue swelling. The case also highlights the importance of a multidisciplinary approach involving anaesthesia, intensive care, and surgical teams in managing such critical cases. 

## Conclusions

CNF is a potentially life-threatening condition that requires prompt diagnosis and aggressive management. AFOI can be a life-saving intervention in the management of airway obstruction caused by this condition. Thorough primary, secondary, and tertiary surveys with appropriate wound care in trauma patients are crucial in preventing hospital-acquired infections that can lead to severe complications.
